# Transient Structural Properties of the Rho GDP-Dissociation Inhibitor

**DOI:** 10.1002/anie.202403941

**Published:** 2024-07-24

**Authors:** Sara Medina Gomez, Ilaria Visco, Felipe Merino, Peter Bieling, Rasmus Linser

**Affiliations:** Department of Chemistry and Chemical Biology, TU Dortmund University, Otto-Hahn-Str. 4a, 44227 Dortmund, Germany; Department of Systemic Cell Biology, Max Planck Institute of Molecular Physiology, Otto-Hahn-Str. 11, 44227 Dortmund, Germany; Department of Protein Evolution, Max Planck Institute of Developmental Biology, Max-Planck-Ring 5, 72076 Tübingen, Germany; Department of Systemic Cell Biology, Max Planck Institute of Molecular Physiology, Otto-Hahn-Str. 11, 44227 Dortmund, Germany; Department of Chemistry and Chemical Biology, TU Dortmund University, Otto-Hahn-Str. 4a, 44227 Dortmund, Germany

**Keywords:** RhoGTPases, NMR spectroscopy, Protein dynamics, Protein complexes, Conformational disorder

## Abstract

Rho GTPases, master spatial regulators of a wide range of cellular processes, are orchestrated by complex formation with guanine nucleotide dissociation inhibitors (RhoGDIs). These have been thought to possess an unstructured N-terminus that inhibits nucleotide exchange of their client upon binding/folding. Via NMR analyses, molecular dynamics simulations, and biochemical assays, we reveal instead pertinent structural properties transiently maintained both, in the presence and absence of the client, imposed onto the terminus context-specifically by modulating interactions with the surface of the folded C-terminal domain. These observations revise the long-standing textbook picture of the GTPases’ mechanism of membrane extraction. Rather than by a disorder-to-order transition upon binding of an inhibitory peptide, the intricate and highly selective extraction process of RhoGTPases is orchestrated via a dynamic ensemble bearing preformed transient structural properties, suitably modulated by the specific surrounding along the multi-step process.

## Introduction

**Figure 1:**
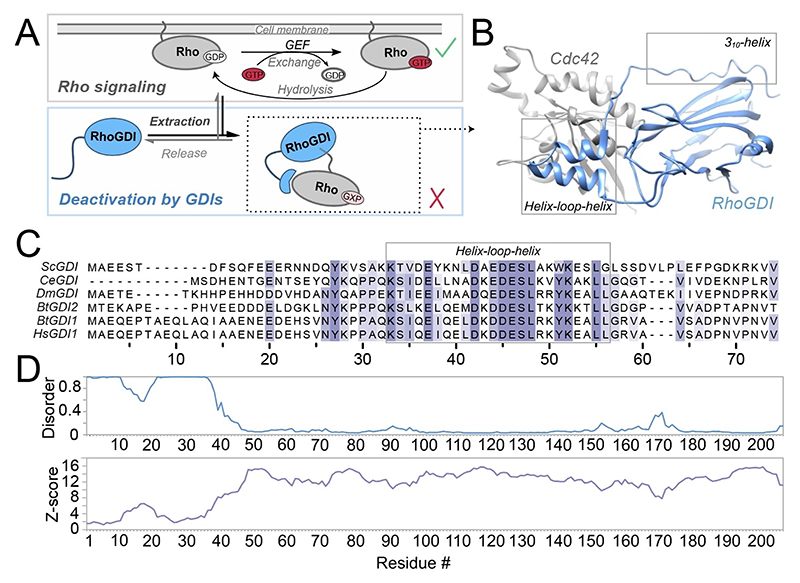
RhoGDI function and bioinformatical analysis. **A)** RhoGDI deactivates RhoGTPases (Rho) by their membrane extraction, upon which a high-affinity complex is formed. “GXP” denotes that both, active (GTP-bound) and inactive (GDP-bound) Rho is extracted.^[2]^ **B)** Crystal structure of RhoGDI in complex with the GTPase Cdc42 (PDB 1DOA), showing the specific tertiary and secondary-structural features newly adopted by the previously flexible N-terminus (gray boxes). **C)** Sequence alignment of the N-terminal region of different RhoGDI orthologs and isoforms (also compare Figure S1). Sc: *Saccharomyces cerevisiae*, Ce: *Caenorhabditis elegans*, Dm: *Drosophila melanogaster*, Bt: *Bos taurus*, Hs: *Homo sapiens*. The color depicts the BLOSUM62 score. L56 and Val74 mark the end of the helix-loop-helix motif and the start of the C-terminal Ig-like domain, respectively. **D)** Disorder prediction (blue) and secondary-structure Z-score (purple) from pure in silico assessment using ODiNPred,^[7]^ predicting a dominantly disordered character of the N-terminal amino acids in consistency with the original literature.^[3b,4,6]^

Small GTPases of the Ras superfamily are key membrane-associated signaling molecules that can assume distinct activity states, which depends on the regulated hydrolysis and exchange of their associated guanine nucleotide. The members of the Rho family of small GTPases, in particular, are responsible for locally modulating cytoskeleton dynamics in intracellular organization, cell polarity, morphogenesis, motility, and other essential cellular processes.^[1]^ Rho guanine nucleotide dissociation inhibitors negatively regulate RhoGTPases by extracting them from membranes,^[2]^ sequestering the membrane-binding carboxy-terminal isoprene moiety^[3]^ and suppressing interactions with regulators of the GTPase activity states such as nucleotide exchange factors (GEFs) or GTPase-activating proteins (GAPs, see Figures 1A and B).^[3–4]^ Thereby, RhoGDIs maintain a large pool of soluble, inactive RhoGTPases. The highly conserved N-terminus of RhoGDI (Figure 1C) is known to be necessary for membrane extraction^[2]^ but has also been thought of as responsible for the inhibition of both nucleotide exchange and hydrolysis by sterically blocking the switch regions of its GTPase clients, hence locking them in an inactive state.^[3a,4–5]^ It also contributes significantly to the binding energy of the RhoGDI:RhoGTPase complex, since its complete removal reduces affinity for GTPases by more than 100-fold.^[4]^ Its functional importance is also in line with the high level of sequence conservation between distinct RhoGDI orthologs and isoforms (Figure 1C). The N-terminus in the apo protein has low binding affinity on its own and has been thought of as an intrinsically disordered protein region lacking defined structural features.^[3b,4,6]^ In particular, no significant NOEs were observed, and the N-terminal resonances cluster in the central region of the NMR HSQC spectra. Figure 1D shows the prediction of order/disorder purely on the basis of insilico assessment using the ODiNPred server.^[7]^

Upon complex formation with GTPases, the GDI N-terminus, by contrast, is currently thought to fold into a structured conformation in an induced-fit manner, now becoming stably bound to both the GTPase and parts of its own folded protein core,^[3a,5]^ see the corresponding crystal structure in Figure 1B. In particular, the folded conformation contains a helix-loop-helix motif, which contacts switch I and II regions of the RhoGTPase, and a partially helical structure for the very N-terminus, including a 3_10_-helix for residues 9 to 16, which folds back against the immunoglobulin (Ig)-like domain that contributes residues to the geranylgeranyl-binding pocket.^[3a]^ This conversion of the secondary structure of the N-terminus from disorder to order is thought to be an integral part of the difficile mechanism to extract RhoGTPases from the membrane. Based on existing structural^[3a,5]^ and biochemical data, a two-step mechanism has been proposed in which the unstructured GDI N-terminus first folds onto the membrane-bound GTPase.^[8]^ A subsequent isomerization event then leads to the swapping of the prenyl moiety between the membrane and the Ig-like domain of RhoGDI.

From the viewpoint of the original literature on RhoGDI structure, the extreme extent of alteration in the structural properties of the RhoGDI N-terminus, to be reconciled with its specific recognition of membrane-bound GTPases, was astonishing. Also, without today’s concepts of intrinsically disordered regions (IDRs) as evolutionarily tuned ensembles of conformers with transient but highly defined characteristics,^[9]^ its character as a disordered peptide in apo GDI, its low binding affinity to the GTPase in isolated form, and the well-defined structural properties in the complex structure, where it folds into a location distant from the nucleotide, seemed surprising. Here we use NMR spectroscopy and biochemical assays to revise the understanding of structural properties for the N-terminal domain of RhoGDI in apo form and when bound to its client, RhoGTPase Cdc42. Given its more widespread expression and higher affinity for its GTPase clients compared to other RhoGDI isoforms,^[3b,10]^ we restrict our analysis to RhoGDI1.

## Results and Discussion

**Figure 2:**
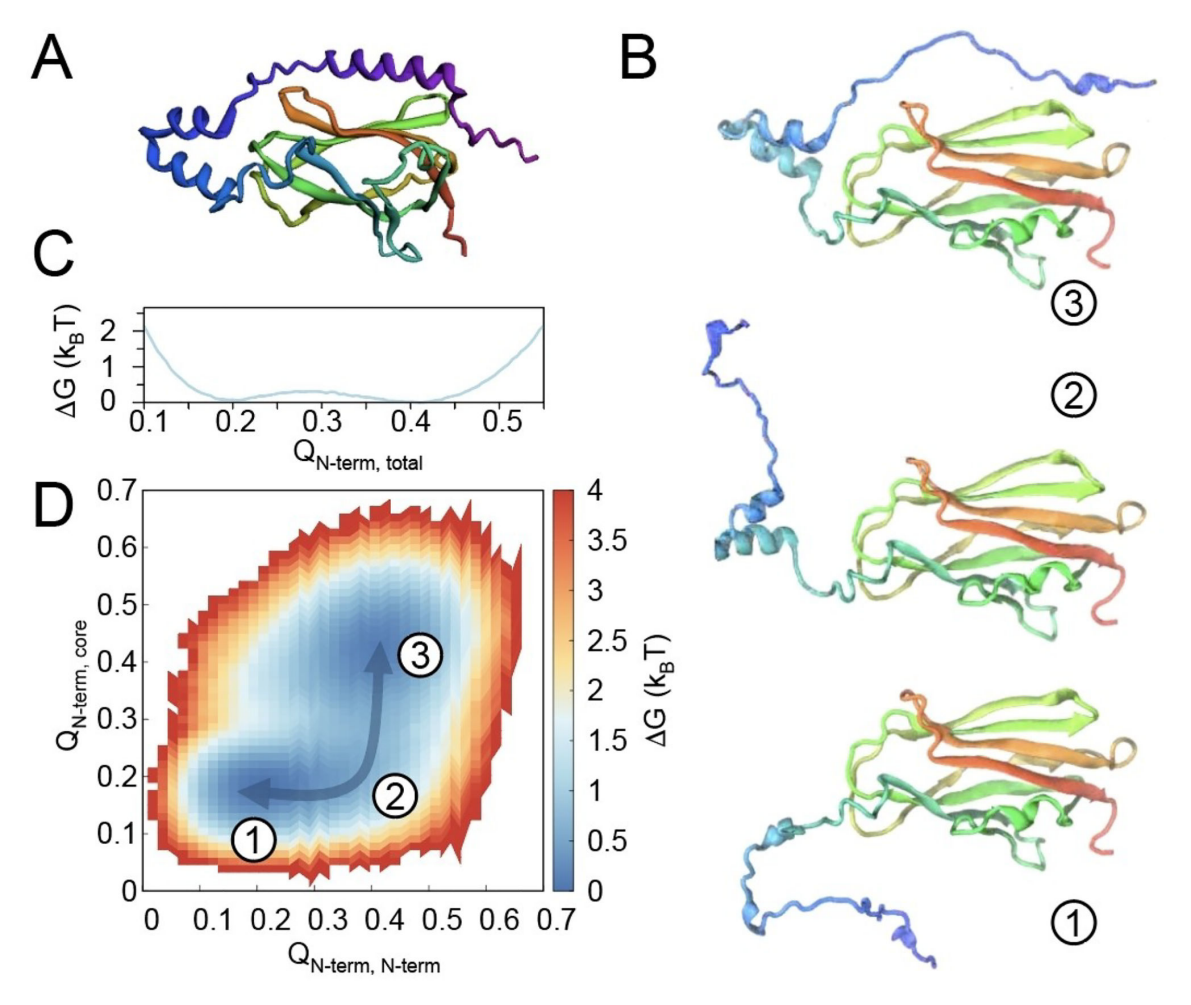
Structural properties from evolutionary analysis and in MD simulations. **A)** Robetta output, predicting strong N-terminal secondary-structural properties instead of the assumed disordered character. **B)** Representative snapshots of conformations visited by RhoGDI during the simulations. (Color in A) and B) according to primary sequence.) **C** and **D)** Folding free-energy profiles for RhoGDI’s N-terminus at its folding temperature: C) Gibbs free energy as a function of the fraction of all native contacts formed by the N-terminus, i.e., amino acids 1–65 (Q_Nterm, total_), and D) 2D free-energy profile of N-terminal folding as a function of the fraction of native contacts among N-terminal atoms (x-axis, Q_N-term, N-term_) and those between N-terminal atoms and the rest of the protein (y-axis, Q_Nterm, core_). The arrow highlights the minimum-free-energy path separating the fully folded from fully unfolded N-terminus.

Our interest in the structural properties of the RhoGDI1N-terminus emerged from an analysis via the current Robetta modeling routine, a deep-learning-based protein structure prediction tool.^[11]^ Even though such prediction results can potentially derive from other than apo states, it sparked our curiosity that the Robetta prediction for the N-terminus of the *apo* protein showed the presences of helical elements for residues 8 to 16 and the helix-loop-helix (Figure 2A), which are not assumed in the current mechanistic model. (Figure S2 compares predictions for the full-length protein with those for an isolated N-terminus.) To test in silico whether/how (partially) folded states are indeed possible within the conformational ensemble of RhoGDI’s N-terminal end and how this could be reconciled with the previous data, we first turned to molecular dynamics (MD) simulations with structure-based potentials. These simplified simulations are known to capture a protein’s folding process without the typical limitations imposed by conventional all-atom MD runs. As a reference for the native state, we used the structure of RhoGDI observed in the complex with Cdc42 (PDBID: 1DOA).

Figure 2B shows three representative conformations visited by the RhoGDI’s N-terminus during the simulations. Consistent with its loose connection to the rest of the protein, the N-terminus explores a large conformational ensemble, while the protein’s core domain remains folded. Folded and unfolded states are separated by a very shallow barrier, well below thermal energy (Figure 2C). Interestingly, we consistently observed conversion between completely unfolded conformations and states where N-terminal secondary-structural elements (in particular the helix-loop-helix motif and the N-terminal 3_10_ helix) are spontaneously formed without attachment to the protein’s core. Indeed, the 2D folding free-energy profile as a function of the intra-N-terminal contacts and those between any N-terminal residues and the core shows that, while the expected free-energy minima representing the fully disordered and the fully attached state are present (states “1” and “3”, respectively, in Figures 2B and D), a shallow path—the minimum free-energy path—connects them through an intermediate with partly folded, but detached N-terminus (state “2”).

**Figure 3:**
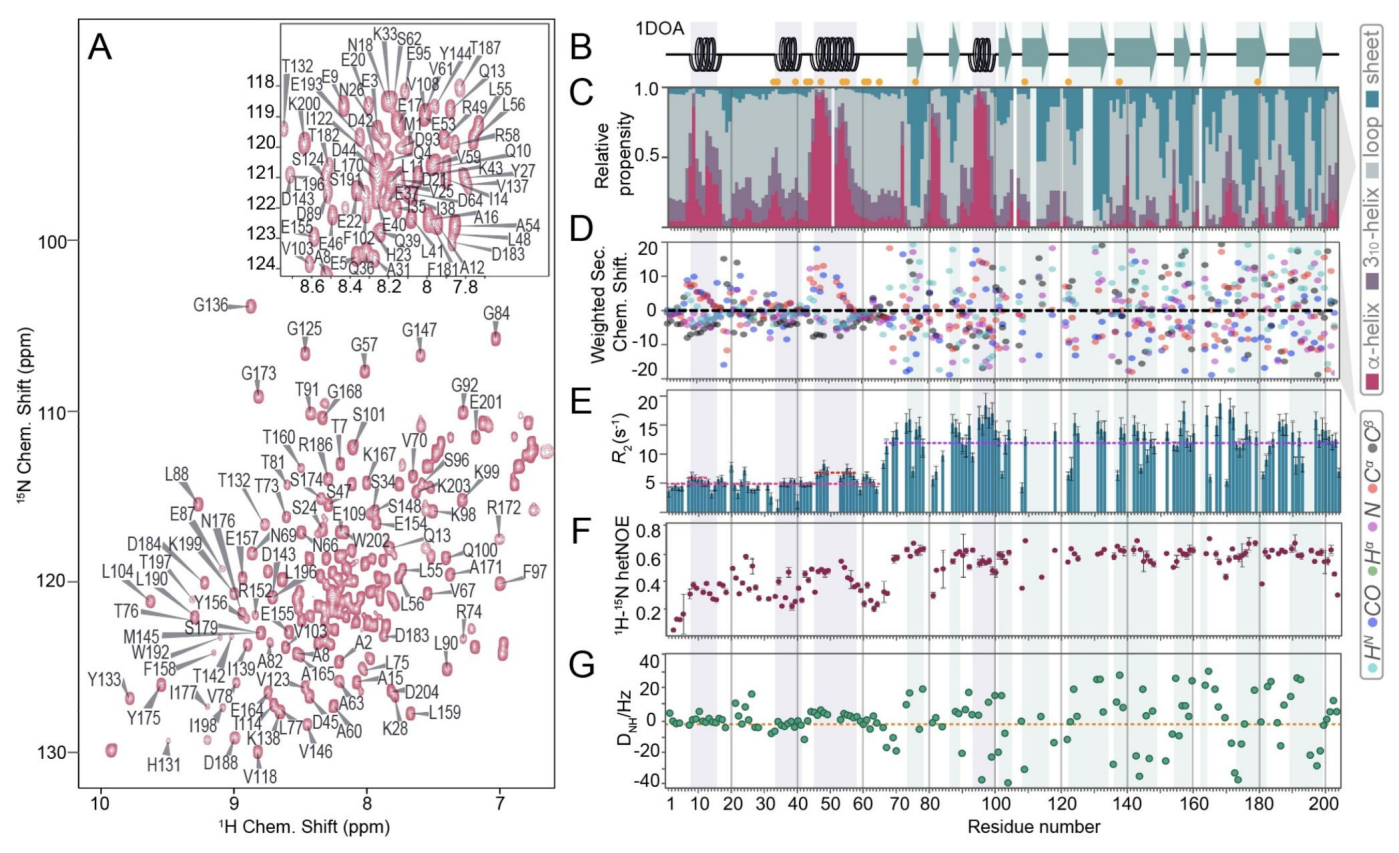
Experimental NMR-based assessment of full-length RhoGDI1 secondary-structural propensity. **A)** Assigned HSQC of the full-length protein recorded at 800 MHz ^1^H Larmor frequency, with parts of the crowded central region only annotated in the magnified excerpt on top. **B)** Secondary structure found in the X-ray structure of the RhoGDI1:Cdc42 complex (PDB 1DOA). **C)** Chemical-shift-based relative secondary-structural propensities (CheSPI) and **D)** neighbor-corrected secondary chemical shifts for H^N^, N, C^α^, C^β^, CO, and H^α^ (drawn in cyan, magenta, red, black, blue, and green, respectively) of apo RhoGDI1. **E)** *R*_2_ relaxation of apo GDI. **F)** ^15^N{^1^H} Steady-state heteronuclear NOE. **G)** Residual dipolar couplings, obtained using 15 mg/mL Pf1 phages, resulting in a ^2^D_2_O quadrupolar splitting of 15 Hz. The color code for secondary-structural analysis in C) is shown on the right. Yellow dots mark residues with partial overlap. All of the above measures consistently suggest existence of local (temporary) secondary structure in part of the N-terminus.

**Figure 4:**
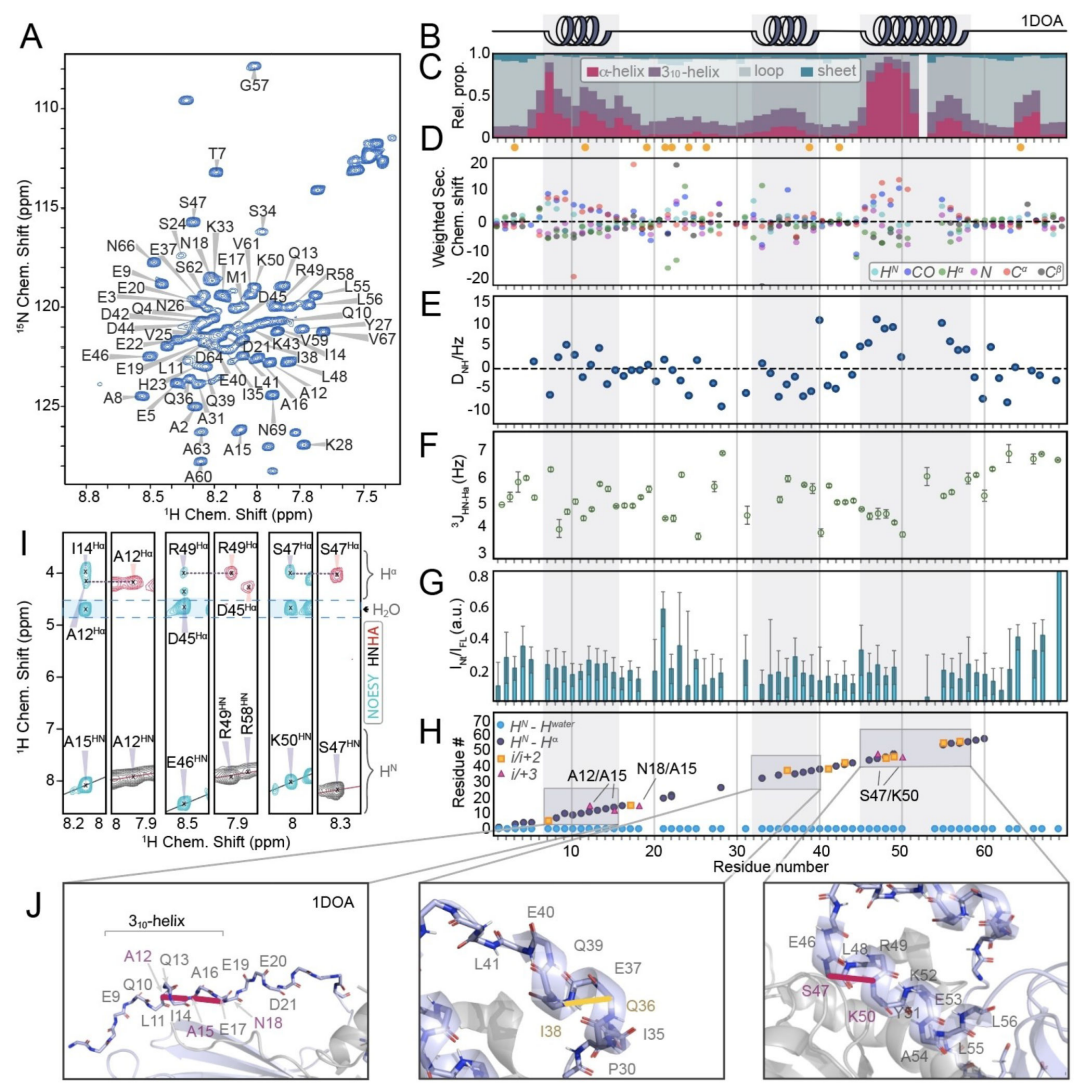
Characterization of the isolated RhoGDI1 N-terminus (here: construct with residues 1–69). **A)** Assigned ^1^H-^15^N HSQC of the isolated N-terminus. **B)** Secondary structure of the X-ray structure (1DOA) of the complex. **C)** Analysis of the extent of secondary structure of the isolated RhoGDI N-terminus from backbone chemical shifts.^[13]^ The color code is depicted on the right. Yellow dots mark residues with partial overlap in 2D (but not in 3D) experiments. **D)** Secondary chemical shifts for H^N^, N, C^α^, C^β^, CO, and H^α^ (drawn in cyan, magenta, red, black, blue, and green, respectively). **E)** Residual dipolar couplings, obtained using 30 mg/mL Pf1 phages, resulting in a ^2^D_2_O quadrupolar splitting of ~25 Hz, with consistently high (positive) values in consecutive residues being typical for helical content within IDPs.^[16]^ **F)** ^3^*J*^HNHα^ couplings as a function of residue, with consistently small values (≲ 4.5 Hz) being typical for helical stretches. **G)** Peak intensity ratio between the isolated N-terminus and the full-length protein as a function of residue. Compare Figs. S10 and S11 for CSPs. **H)** Presence of amide NOE cross peaks in a 3D ^15^N-edited NOESY experiment, showing the presence of transient secondary structure via magnetization transfer from amide protons to close-by H^α^ spins (*i* to *i*+2 and *i* to *i*+3 contacts shown by yellow squares and magenta triangles, respectively, contacts to water by blue circles in the bottom). **I)** Exemplary strips from a 3D ^15^N-edited NOESY (cyan) with important contacts, in conjunction with matching strips from an HNHA experiment (red/black). **J)** Depiction of some NOE contacts, denoting temporary secondary structure, in the context of matching secondary structure seen in the crystallographic complex, magenta and yellow dashed lines denoting experimentally observed *i* to *i*+3 (left and right panel) and *i* to *i*+2 (center panel) H^N^-to-H^α^ contacts, respectively.

To quantify the tendency towards a preformed N-terminal binding interface for GDI/GTPase interactions experimentally, we turned to NMR spectroscopic characterization of residue-specific residual structural and dynamics properties.^[12]^ For that purpose, three different constructs of bovine RhoGDI1 were expressed recombinantly and purified according to standard procedures. In particular, full-length RhoGDI1 (residues 1 to 204) was produced in triply labeled (^2^H, ^13^C, ^15^N) fashion, whereas two isolated N-terminal fragments (residues 1 to 59 and residues 1 to 69) were expressed in doubly, ^13^C, ^15^N-labeled fashion. (See details on expression and purification in the Materials and Methods, SI.) To enable site-specific characterization of the secondary structural features, a comprehensive suite of 3D solution NMR spectra of the different RhoGDI1 constructs were recorded. (See details in the Experimental Section.) As the N-terminal residues were known to cluster in a heavily overlapped central region of the H/N plane, compromising the assignment of the core residues in previous studies,^[4]^ we first turned to the assignment of these residues in the isolated N-terminal fragments. Complete assignment of the N-terminal residues, their successive transfer to the full-length protein sample, and comprehensive assignment of the remaining residues in the full-length construct thus became possible. (See the Supporting Information for the backbone assignments used for this purpose.) Figures 3A and 4A show the assigned HSQC spectra for the entire RhoGDI1 protein and the (longer) isolated N-terminus, respectively. Chemical shifts were deposited into the BRMB under accession number 51835.

**Figure 5:**
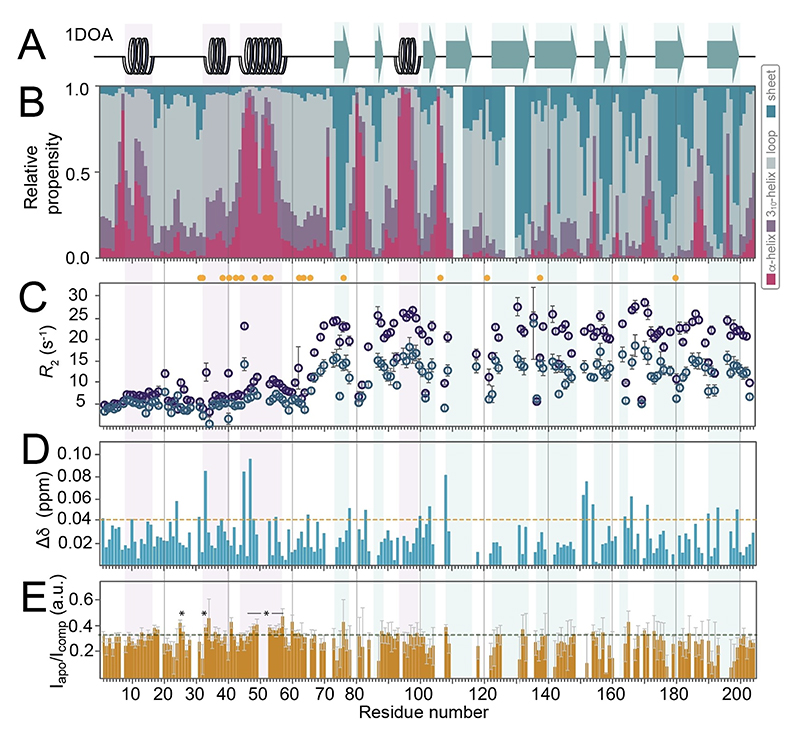
Structure and dynamics of the RhoGDI1N-terminus in the GDI:Cdc42 complex. **A)** Secondary structure of the complex seen in 1DOA. **B)** Secondary-structural propensities of the complex derived from experimental chemical shifts (CheSPI). **C)** *R*_2_ rates of the complex (cyan), overlaid with those of the apo form (dark blue). Compare Figure S12 for *R*_1_ and *R*_2_/*R*_1_ values. **D)** Chemical-shift perturbations upon complex formation. Yellow dots mark partial overlap in pseudo-3D relaxation data (not, however, in the 3D-based chemical-shift data). **E)** Peak intensity ratio between the apo protein and the RhoGDI: Cdc42 complex as a function of residue. The dashed line has been included for the discernment of residues with slightly increased ratios.

The chemical-shift assignments provided the basis for experimental interrogation of RhoGDI1 structural features in a site-specific manner in solution. We first used the computational framework CheSPI (Chemical shift Secondary structure Population Inference,^[13]^ Figure 3C), engineered to quantify relative structural features in both, ordered and disordered systems based on neighbor-corrected chemical shifts, for secondary-structural assessment based on actual backbone ^1^H^N, 15^N, ^13^CO, ^13^C^α^ and ^13^C^β^ chemical shifts. (The underlying individual, nucleus-specific secondary chemical shifts, calculated according to Nielsen et al.,^[14]^ are plotted in Figure 3D; respective assessments using Talos+^[15]^ and the Z-score obtained from CheSPI analysis, indicating the level of order/disorder as a function of sequence, are shown in Figures S3 and S4, respectively.) The obtained results confirm the presence of pronounced secondary structure within the N-terminus of the apo form of the full-length protein. In particular, residues 42 to 55 display a stable helical fragment, exactly matching the helical structure found for the crystallographic RhoGDI: GTPase complex.^[3a,5]^ The second, shorter helical stretch seen in the X-ray structure, around residues 32 to 40, also shows helical secondary structural propensity by NMR, however, with much weaker helical (around 30% overall) propensity and a lower Z-score than the first one. A third helical fragment is found experimentally for residues 8–15 (the 3_10_-region in the crystallographic complex), again with a quantitatively lower propensity (around 50%) and moderate Z-score. All of the (transient) helical propensities in the terminus align with the structural features of RhoGDI1 in the complex (Figure 3B). Figure 3E shows site-specific *R*_2_ rates of the apo-RhoGDI1. (Note that in contrast to the well-resolved 3D triple-resonance spectra, in H/N-based readouts, part of the residues show partial overlap, as marked by yellow dots in Figures 3–5.) These rates (around 5 s^−1^, as opposed to rates of around 15 s^−1^ for the structured residues in the core) confirm a greater overall flexibility (detachment and an individual tumbling correlation time) of the N-terminus, however, with slight but unequivocal elevations (7–8 s^−1^) for the transient secondary structure in the very N-terminus (around residue 8) and in particular for the more durable helix of the helix-loop-helix motif (around residue 50). The significant difference compared to *R*_2_ rates generally found for the core shows that none of the elements in the terminus with transient secondary structure firmly associates with the core. Unambiguous confirmation of a partially maintained helicity in the context of high overall flexibility is also obtained from ^15^N{^1^H} steady-state hetero-nuclear NOEs (Figure 3F) and residual dipolar couplings (using Pf1 phages), with slightly positive values in consecutive residues being typical for helical propensity within intrinsically disordered regions^[16]^ (Figure 3G, see the Supporting Information for preparative details).

To decipher whether/to what extent the residual secondary-structural propensities found for the N-terminus are fully intrinsic properties or further shaped context-specifically by local interactions, an assessment of secondary-structural features was performed for two isolated N-terminal constructs (Figure 4) as (unphysiological) reference cases. (Note that the opposite case, the assessment of a truncated core RhoGDI in comparison with the full-length version, can be found in Gosser et al.^[4]^) CheSPI results (Figures 4C and S5) still qualitatively agree with the propensities of the full-length protein. (The underlying secondary chemical shifts for individual types of nuclei are shown in Figure 4D, comparisons of CheSPI and TALOS are shown in Figs. S5 and S6, and disorder prediction from pure in silico assessment using ODiNPred^[7,13]^ is shown for comparison in Figure S7.) Like in the full-length protein, the helical stretch seen for residues 8–15 in the longer construct has similarly low scores (slightly higher than in the full-length protein, but with an average still not exceeding 0.5), and transient helical properties are again found around residues 32–40 and 45–55. The *quantitative* extent of helical propensity of the latter regions (especially residues 32–40 and 50–55), however, differs, with only minor degrees of helicity in the isolated terminus (around ~30 and 50%, respectively, compared to around 60 and 90%, respectively, in the full-length protein). As for the full-length GDI, the partially maintained helicity is qualitatively confirmed by residual dipolar couplings, measured using 10–30 mg/mL Pf1 phages (Figure 4E). In addition, ^3^*J*^HNHα^ couplings (Figure 4F and Figure S8) and the occurrence of typical NOE patterns between amide and H^α^ protons (Figure 4H–J) were probed, which unambiguously confirm the transient formation of helical structure where expected. Figure S9 adds Redfield type relaxation data *R*_1_, *R*_2_, and ^15^N{^1^H} steady-state heteronuclear NOEs, which are again congruent with the above. The above-mentioned quantitative differences between the isolated N-terminus and the full-length protein for part of the helical stretches of the final binding interface to the GTPase suggest that (transient) intra- and intermolecular interactions further modulate the structural propensities, whereas the context of a core aids in prestructuring the GDI interface for the initiation of complex formation: Whereas on its own, most of the C-terminal residues of the N-terminus are dominated by the features of an intrinsically disordered protein and show only little tendency to form the final interface, in line with the previously observed inability of the isolated N-terminus to capture the client,^[4]^ the interactions of the N-terminus with the core—albeit not firmly attached—further increase the population of transient secondary structural features, such that the interface for GTPase binding becomes preformed more substantially. The variability of secondary structure in the helix-loop-helix motif, as opposed to a stable intrinsic property, also manifests itself in the quantitative differences in residual secondary structure between the shorter and the longer N-terminal construct (compare Figure S5 vs. Figure S6). The effect is confirmed by differences in chemical-shift perturbations (CSPs) between the two isolated N-terminal constructs with respect to full-length GDI (Figs. S10 and S11), together confirming a general context dependency of the structural features in both helices of the helix-loop-helix motif. To complement CSPs, in which contributions from secondary-structure modulation are convoluted with proximity-based shift changes, we also probed changes in signal intensities compared to the full-length protein (Figure 4G). Here, elevated values suggest that in the full-length protein the respective residues are either more restricted (expected and observed towards the new C-terminus) or experience exchange broadening (residues 20–24), probably due to transient contacts to the core. Both of these trends are also found for the CSPs with respect to the full-length protein (see Figure S11). Paramagnetic labels would be interesting to probe intra- and inter-domain interactions more sensitively. Note, however, that apart from the preparative challenges for this system, these may also introduce chemical and steric changes that on their own might distort the sensitive balance between order and disorder.

We next compared the transient structural properties that we observed in the apo protein with those of RhoGDI1 in complex with its binding partner GTPase Cdc42 (Figure 5). For this purpose, triple-labeled RhoGDI was quantitatively incorporated into a stoichiometric 1:1 complex by addition of excess (isotopically unlabeled) Cdc42, which had been homogeneously geranylgeranlyated in vitro. Given the sub-nM affinity of the complex (see below), the heterodimeric complex could readily be separated from excess free Cdc42 by size exclusion chromatography, which avoids detergents in the successive experiments. (See Materials and Methods for details of these procedures.) In order to verify complex formation, the GDI *R*_2_ rates from the two samples were compared (Figure 5C). *R*_2_ rates are modulated by the time scale of molecular tumbling (τ_c_), and an overall increase is expected when the effective molecular weight and thus τ_c_ increase. This tendency is clearly observed both, for the core residues (from 11.9±3.9 to 19.6±5.8) as well as for the N-terminus (from 5.2±2.0 to 7.7±3.1) when overlaying the *R*_2_ rates from both samples. Also compare Figure S12 for *R*_2_/*R*_1_ ratios as well as *R*_1_ rates as a representative of effective local correlation times and fast motion, respectively.

Even though the X-ray structure of the complex (PDB 1DOA) converges to a defined structure of the N-terminus, the electron density already reveals a certain degree of flexibility even under cryogenic conditions in the crystal (compare Figure S13). C^α^ B-factors, reaching down to 34 within the remainder of the structure, bear values well above 100 up to residue 25 and between 59 and 66. Representing closer-to-physiological conditions in solution, the NMR assessment at room temperature now reveals a highly mobile behavior of the N-terminus—even in the complex. The increase in *R*_2_ rates of representative residues in the inside of the core region agrees with what is expected for a complex of 46.6 kDa molecular weight at 25 °C (see Figure 5C). In addition to the systematic overall increase of *R*_2_ rates of the N-terminal residues (from residue 7 onwards), a stronger increase is observed for those stretches with higher relative secondary-structural propensity (to well above 10 s^−1^ for the residues around residue 50). The largely retained dynamic behavior contradicts the stable folding onto the GTPase assumed in the bound state hitherto and rather demonstrates such interactions with the client that retain a high degree of conformational freedom in the N-terminus. Adding to this picture of a rather loose association of the N-terminus and its preformed structural elements, only minor secondary-structural changes upon complex formation are apparent (Figure 5B, compared to Figure 3C). (The individual secondary chemical-shift values are represented in Figure S14.) Apart from the moderate increase in most N-terminal *R*_2_ rates, weak local chemical-shift perturbations are observed (e.g., for residues K33, D45, and S47, Figures 5D and S15) upon complex formation, matching with the expected N-terminal interactions between Cdc42 and RhoGDI1.^[3a]^ In line with these observations, these sites tend to show a particular increase of the *R*_2_ rates upon complex formation (Figure 5C), likely due to chemical-exchange contributions associated with these temporary contacts. Figure 5E also depicts intensity ratios between the apo form and the complex. Asterisks denote residues with slightly increased ratios, which align with regions where temporary contacts might impact secondary-structural and flexibility features. (Note that intensity ratios are to be taken with care as they depend on multiple, convoluted properties.) Together, these observations suggest a loose, plastic interaction of the GDI N-terminus with the binding partner and the remainder of the complex, with a remaining flexibility of most N-terminal residues, weak specific contacts, and the intrinsic secondary-structure distribution further maintained upon intermediate-timescale association/dissociation dynamics. Further characterization of the transient intermolecular contacts between the GDI N-terminus and Cdc42 would also be interesting from the client side. This is currently limited, however, by the low compatibility of the required geranylation of Cdc42 with either, isotope labeling/chemical-shift assignment or paramagnetic spin labeling.

**Figure 6:**
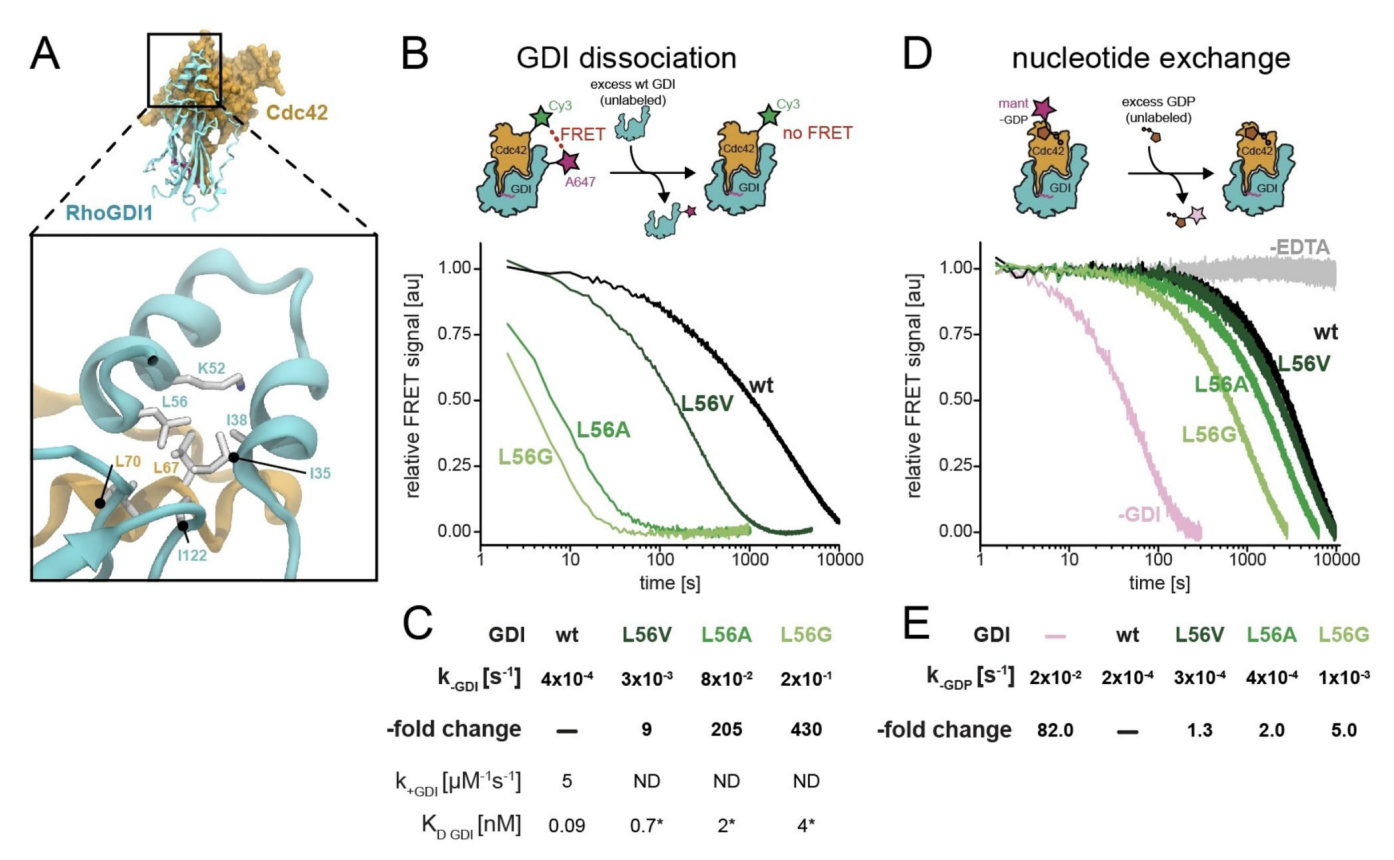
Assessment of the modulation of complex stability by destabilization of intramolecular interactions in the RhoGDI N-terminus via FRET. **A)** Environment of L56 within the overall structure (top) and locally within the C-terminal end of the helix-loop-helix motif (bottom). **B)** Top: Scheme of the FRET-based dissociation experiment. A 1 : 1 complex between Cy3-labeled, prenylated Cdc42 and Alexa647-labeled RhoGDI1 (wt or mutant) is mixed with excess wt RhoGDI1. The dissociation of the complex is followed over time by the loss in FRET between the two fluorophores. Bottom: Decrease in FRET signal as a function of time for wt RhoGDI (black) and mutants L56V, L56A, and L56G (dark to light green) as indicated. **C)** Summary of the dissociation rates (*k*_-GDI_) of GDI (either wt or mutants as indicated) from prenylated Cdc42 obtained from mono-exponential fits to the data shown in B and the relative rate enhancement compared to wildtype GDI. The corresponding association (*k*_+GDI_) rate was measured for wildtype GDI (see Figure S17) and assumed to be the same or slower for the GDI variants. Equilibrium dissociation constants (*K*_D GDI_) were calculated by the rate of the measured dissociation rate constants and the measured or assumed association rate constant. **D)** Top: Scheme of the fluorescence intensity-based nucleotide exchange experiment. Prenylated Cdc42, loaded with Mant-GDP, was mixed with excess (5 μM) RhoGDI1 (wt or mutant) to ensure continuous RhoGDI binding during the experiment. Nucleotide dissociation was initiated by the addition of excess unlabeled GDP (100 μM) and followed over time by the loss in fluorescence intensity due to solvent exposure of the Mant-GDP upon unbinding from Cdc42 in complex with RhoGDI. Bottom: Decrease in Mant fluorescence as a function of time for either free prenylated Cdc42 (pink) or in complex with RhoGDI (either wildtype (black) or mutants L56V, L56A, and L56G (dark to light green)) as indicated. **E)** Summary of the nucleotide exchange rates (*k*_-GDP_) of Mant-GDP from prenylated Cdc42 either free or in complex with RhoGDI (either wt or mutants as indicated) obtained from mono-exponential fits to the data shown in D.

Finally, we aimed to assess biochemically the importance of the internal structural stability of the GDI N-terminus for enhanced GTPase binding. On the basis of the above MD simulations and NMR data, we searched for a residue that is not part of the binding interface itself—and hence does not impact the binding affinity directly through intermolecular interactions with Cdc42—but would only impact the structural stability of the temporary helix-loop-helix structural element within the GDI N-terminus. (A concomitant detrimental impact on *secondary*-structural free energies, not experimentally elucidated for the mutants in the following, is likely and no drawback.) To identify our best-candidate residue, we performed an alanine-scanning of the interface using the Robetta server^[17]^ (see Figure S16). Figure 6A visualizes the embedding of residue L56 in the helix-helix interactions via hydrophobic contact with I35. We chose decreasingly conservative mutations from Leu56 to Val, Ala, or Gly and read out the kinetic stability of the complex by a Förster resonance energy transfer (FRET)-based dissociation assay. In brief, the FRET signal of a dual-labeled complex (80 nM) with an intermolecular FRET pair (an Alexa647 acceptor on RhoGDI1 and a Cy3 donor on Cdc42, respectively) decreases upon mixing with excess (5 μM) unlabeled GDI, hence reporting on complex dissociation as a function of time. Indeed, we observed a strong and consistent increase in the dissociation rate constant of the complex upon perturbation of the inter-helical interaction with decreasing side chain length (Figure 6B,C). These measurements, together with the association rate constant we independently determined for wildtype GDI (*k*_+GDI_ = 5 s^−1^ μM^−1^, Figure S17), allowed us to estimate the changes in complex affinity (Figure 6C). We assumed that the GDI mutations cannot majorly impact the fast association rate constant for complex formation, which (for molecules in this molecular-weight regime) falls close to the diffusion limit and therefore cannot become much faster. Hence, we observed as previously^[18]^ that wildtype GDI bound its GTPase clients with extremely high affinity (*K*_D_ = 90 pM) (Figure 6C). Mutations of L56 lead to a progressive loss in affinity up to more than 400-fold. However, even the most weakly binding GDI variant (L56G) still possessed high affinity (*K*_D_ = 4 nM) for prenylated Cdc42.

One established function of the GDI N-terminus is the inhibition of nucleotide exchange and hydrolysis in its bound GTPase.^[3a,4–5]^ Hence, we wondered how the intrinsic N-terminal dynamics and their enhancement by the destabilizing mutations in GDI affect the kinetics of GTPase nucleotide exchange. To this end, we measured EDTA-induced nucleotide dissociation using the fluorescently-labeled GDP analogue Mant-GDP bound to prenylated Cdc42 and in the presence of excess unlabeled GDP in solution (Figure 6D). We conducted these experiments in the presence or absence of saturating (5 μM) concentrations of RhoGDI (either wildtype of mutants) to generate the fully GDI-bound or GDI-free state during the experiment. We observed that binding of wildtype GDI strongly (83-fold) inhibited nucleotide exchange in Cdc42 as expected (Figure 6D). However, the small remaining rate of nucleotide exchange (Figure 6D, *k*_-GDP_ = 2×10^−4^ s^−1^) was similarly slow as GDI dissociation (*k*_-GDI_ = 4×10^−4^ s^−1^), showing that these processes occur on a similar time scale for wildtype GDI. This means the pronounced N-terminal dynamics we observe for GDI within the complex are orders of magnitude faster. (The N-terminus has a shorter correlation time than the protein core, a property on the ns timescale.) The proposed steric blockage of nucleotide exchange by the N-terminus, an inherently slow process (*k*_-GDP_ = 2×10^−2^ s^−1^ even in the absence of GDI), could hence be explained solely as an ensemble property, where access to the binding site is partly reduced due to the presence of multiple (variable) conformations close to the binding site, not by creation of a firm steric blockage. We found that destabilizing the N-terminal structure through mutations of L56 resulted in a (up to 5-fold) acceleration of nucleotide dissociation in proportion to the severity of the perturbation (Figure 6D/E), in congruency with the decreased lifetime of the GTPase:GDI complex. Whereas any “blocking” properties of the ensemble may further be modulated by the mutation, the congruency of rates between these processes suggests that nucleotide exchange is indeed abolished during the lifetime of the protein:protein complex (modulated by the interaction with the terminus). (The discrepancy between the larger acceleration of the protein complex dissociation, with rates up to 2×10^−1^ s^−1^, compared to a smaller acceleration of nucleotide exchange, up to 1×10^−3^ s^−1^, results from the fact that acceleration of nucleotide exchange by mutation is convoluted with the exchange rate in free Cdc42 of maximally 2×10^−2^ s^−1^.) Instead of by a classical (i.e., static) steric blocking, for which sufficiently long-lived direct interactions between the GDI N-terminus and the nucleotide binding site can be ruled out, the known effect of nucleotide exchange being effectively slowed down by the GDI could be due either to highly repulsive effects of the dynamic ensemble. However, the congruency of life-times could also be speculated to derive from other, more *overarching* features of complex formation, for example, a modulation of *GTPase internal dynamics* upon GDI binding (e.g., stalling of breathing motion, otherwise facilitating exchange in the free GTPase case) as long as the complex persists—or even a combination of mechanisms.

The behavior witnessed in the assessment of the structural properties of the RhoGDI N-terminus by NMR spectroscopy differs from the long-standing assumption of a disorder-to-order transition upon complex formation with its GTPase client. Instead, in full-length RhoGDI in its apo state, the N-terminus contains most of the architecture stably adopted in the X-ray structure of the complex as preformed structural elements. Conversely, a high degree of flexibility remains in the N-terminal residues even after complex formation. This is true even though expected chemical-shift perturbations and an overall increase of *R*_2_ rates unambiguously confirm the formation of the heterodimeric complex. Even though at this point, it is unclear what these peculiar structural properties are needed for in the cellular context, it becomes clear that the prevalent model of RhoGDIs and the interaction with their clients has to be adapted, matching with an emerging general picture of proteins as dynamic conformational ensembles with heterogeneously defined structural properties. In particular, for intrinsically disordered regions, site-specific transient secondary structure is assumed to bear important effects on the binding properties, affinity, and selectivity towards binding partners.^[19]^ Adding to folding/binding^[21]^ as rather gradual changes within differentially skewed equilibria, the heterogeneous free-energy landscapes of dynamic ensembles also allow fine-tuning of structural properties as a function of external events, which can sensitively influence downstream signaling. Whereas mechanistic data in the biological context were not pursued in this study, the presence of transient and externally tunable structural features in the RhoGDI N-terminus can be speculated to serve a more complex mechanistic role compared to the previously assumed, binary disorder-to-order transition needed for steric inhibition of nucleotide exchange. Overall flexibility despite tunable local pre-ordering might be mechanistically favorable for membrane extraction of GTPases by RhoGDI, likely a multi-step and reversible process^[8]^ that in fact seems reconcilable neither with a high-affinity yet fully rigid nor a fully unstructured but low-affinity N-terminus. The context dependency of structural propensities of the N-terminal binding interface observed in the presence (full length GDI) or absence of interactions with surfaces of the core (isolated N-termini) and with differential lengths of the terminal constructs furthermore point to a *modulation* of their folding energy landscape by transient intra- or inter-domain and intermolecular interactions, loosely reminiscent of cooperative folding of nearby individual domains^[22]^ or modulation of free-energy surfaces by crowding agents and other surface interactions in a cellular environment.^[23]^ One could speculate that this property might improve fast and selective binding of the client upon first contact while maintaining compatibility with other steps of the process. Overall, the fine-tuned transient-structural and dynamic properties observed for the N-terminal sequence are in line with its high degree of sequence conservation, which has been note-worthy in the light that only a small fraction of the residues are thought to undergo direct interaction with the client.

Speaking for a well-balanced co-existence of structure and disorder, dynamically interchanging in a context-specific equilibrium, the data effectively reconcile the various insights for RhoGDIs and their GTPase interactions from past work. The RhoGDI case adds to the growing spectrum of cellular contexts found to hinge on such co-existence, of importance in a range of different areas of structural biology^[12b,24]^ and bearing peculiar opportunities both, for nature and upon human interference to tweak affinity and selectivity.

## Conclusion

Here, we used NMR spectroscopy in solution to capture the inherent structural properties of the N-terminus of the Rho guanine nucleotide dissociation inhibitor in the absence and presence of its GTPase client. Opposed to a binary disorder-to-order transition upon complex formation assumed so far, the data demonstrate a context-specific propensity of the binding interface to partially preform those structural features later observed in the binding of the RhoGDI to its GTPase already in apo form. Conversely, the overall degree of flexibility for this domain remains high even upon complexation. Together with FRET-observed dissociation assays, the data point to a fine-tuned coexistence of order and disorder for the N-terminus, forming a well-tuned ensemble of transiently adopted structures. Even though the actual cellular implications of these properties are beyond the insights achievable by the NMR experiments, the tunable dynamic interplay partly revises and reveals remaining gaps in the current understanding of GDIs’ capturing and tethering of clients.

## Supplementary Material

SI

## Data Availability

The data that support the findings of this study are openly available in BMRB at https://bmrb.io/, reference number 51835.
