# Supplementary material for: Transient Structural Properties of the Rho GDP-Dissociation Inhibitor
Source: Angew Chem Int Ed Engl. Author manuscript; Available in PMC 2024 Sep 9. (PMC7616425; doi:10.1002/anie.202403941)
Supplement: SI [file EMS198466-supplement-SI.pdf]

Angewandte Chemie
Eine Zeitschrift der Gesellschaft Deutscher Chemiker

Supporting Information

Transient Structural Properties of the Rho GDP-Dissociation Inhibitor

S. Medina Gomez, I. Visco, F. Merino, P. Bieling, R. Linser**

Supporting Information for

Transient Structural Properties of the Rho GDP-Dissociation Inhibitor

By Sara Medina Gomez[1], Ilaria Visco[2], Felipe Merino[3], Peter Bieling[2]*, Rasmus Linser[1]*

[1] Department of Chemistry and Chemical Biology, TU Dortmund University, Dortmund, Germany.
[2] Department of Systemic Cell Biology, Max Planck Institute of Molecular Physiology, Dortmund, Germany.
[3] Department of Protein Evolution, Max Planck Institute of Developmental Biology, Tübingen, Germany.

Materials and Methods

Expression and purification of recombinant proteins

BL21(DE3) chemically competent *E. coli* cells were transformed with GST-Pre-RhoGDI1 (bovine, either full length including an N-terminal cysteine residue for chemical labeling or the isolated N-terminus (residues 1-59 or 1-69) in a pGEX6P vector), induced with 250µM IPTG and incubated at 30°C for 5h. For NMR experiments, M9 minimal medium with 2g/L $^{2}H_{7}{}^{13}C_{6}$ D-glucose, 1g/L $^{15}NH_4Cl$ and D_2O was used. Bacterial cells were harvested, centrifuged at 4000 x g for 20 min, and pellets flash frozen in liquid nitrogen and stored at -80°C. Frozen pellets were resuspended in a 3x volume of lysis buffer (50mM KP_i pH 8, 400 mM KCl, 1 mM EDTA, 5 mM β-ME, 1 mM PMSF, 1 mM benzamidine) and lysed with a high-pressure homogenizer at 4°C. Lysate was cleared by centrifugation at 100,000 x g for 1 hr and applied to a glutathione sepharose 4 fast flow column bed (GE-Healthcare, Chicago, IL) equilibrated with wash buffer (50 mM KP_i pH 8, 400 mM KCl, 1 mM EDTA, 5 mM βME, 1 mM benzamidine). The column was washed with wash buffer, and protein was eluted with elution buffer (50 mM KP_i pH 8, 400 mM KCl, 1 mM EDTA, 5 mM β-ME, 1 mM benzamidine, 10 mM reduced L-glutathione). Peak fractions were pooled, protein concentration was estimated with Bradford assay (Bio-Rad Laboratories, Inc, Hercules, CA), and PreScission protease was added at 1:30. After ON dialysis in wash buffer at 4°C, the sample was recirculated on the same glutathione sepharose 4 fast flow column bed re-equilibrated in wash buffer. Flow-through was collected, concentrated and gel filtered on a HiLoad Superdex 200 or 75 pg column (GE-Healthcare) in storage buffer (20 mM HEPES pH 7.5, 150 mM KCl, 0.5 mM TCEP, 20% Glycerol). For fluorescent labeling, RhoGDI1 was incubated with a 5-fold molar excess of Alexa647-Maleimide for 1h on ice, followed by quenching by the addition of 2mM DTT and an additional round of gel filtration over a Superdex 75 10/300 GL column (GE-Healthcare). Peak fractions of either unlabeled or Alexa647-labeled RhoGDI were pooled, concentrated, and either flash frozen in liquid nitrogen and stored at −80°C or directly used in NMR experiments. Protein purification and purity were determined by Coomassie stain of 12% SDS-PAGE, protein concentration was determined by absorbance at 280 nm.

His_6-zTag-TEV-Cdc42 (human, full length, codon optimized for *E. coli* expression) in a pETMz2 vector was expressed and purified similarly to GDI with the following differences: (1) Proteins were expressed with 1 mM IPTG at 37°C for 4hrs; (2) the affinity step was performed on HiTrap Chelating HP columns loaded with Co^{2+} and equilibrated in 50 mM HEPES pH 7.5, 50 mM NaCl, 5 mM $MgCl_2$, 0.5 mM β-ME, 100 µM ATP and 100 µM GDP; (3) the protein was gel filtered in storage buffer (50 mM HEPES pH 7.5,

50 mM NaCl, 2 mM $MgCl_2$, 2 mM DTT, 20% glycerol).The RabGTTase beta subunit was expressed and purified as described before[1].

Fluorescent N-terminal labeling or nucleotide exchange with Mant-GDI and prenylation of Cdc42 and complex formation with RhoGDI1

Cdc42 was *in-vitro* prenylated and labeled as previously described[2]. Purified Cdc42 was mixed with geranylgeranyl transferase type 1 and geranylgeranyl diphosphate at 10:1:30 ratio in prenylation buffer (50 mM HEPES pH 7.5, 50 mM NaCl, 2 mM $MgCl_2$, 2 mM DTT, 200 µM GDP, 2% CHAPS), and incubated ON on a rotating mixer at 4°C. The sample was spun in a TLA-100 rotor (Beckman Coulter, Brea, CA) at 80,000 rpm for 30 minutes at 4 °C and gel filtered on a HiLoad Superdex 75pg column (GE-Healthcare) equilibrated with prenylation buffer with 0.5 % CHAPS. Peak fractions were pooled, concentrated and buffer exchanged in prenylation buffer without CHAPS in the presence of equimolar concentration of isotope labelled RhoGDI1 on a NAP-5 column (GE-Healthcare). The complex was further concentrated and directly used for NMR experiments.

For the FRET-based association and dissociation assays, Cdc42 was labeled at the N-terminus with Cy3 using sortase-mediated peptide ligation. Prenylated Cdc42 was incubated with sortase and Cy3 N-terminally labeled LPETGG peptide at 3:1:8 ratio and incubated ON at 16°C. This sample was then gel-filtered on a HiLoad Superdex 75pg column (GE-Healthcare) equilibrated with prenylation buffer with 0.5 % CHAPS. Fractions corresponding to prenylated Cdc42 were pooled, concentrated and mixed with a 1.2-fold molar excess of the RabGGTase beta subunit and buffer exchanged in prenylation buffer without CHAPS and containing 20% glycerol on a NAP-5 column (GE-Healthcare). Residual detergent was removed by Pierce Detergent Removal Spin Column (Thermo Fisher, Carlsbad, CA). RabGTTase beta acts a weakly-binding chaperone for the geranylgeranyl moiety, which is otherwise insoluble in the absence of detergent. Proteins were concentrated and flash frozen in liquid nitrogen and stored at -80°.

For nucleotide exchange experiments, unlabeled, prenylated Cdc42 was loaded with a 10-fold molar excess of Mant-GDP (Jena Biosciences) by the addition of EDTA (10mM), incubated for 30min at room temperature followed by the addition of excess $MgCl_2$ (20mM) and additional incubation for 30min on ice. Samples were then mixed with a 1.2-fold molar excess of the RabGGTase beta subunit and buffer exchanged in prenylation buffer without CHAPS and containing 20% glycerol on a NAP-5 column (GE-Healthcare). Residual detergent was removed by Pierce Detergent Removal Spin Column (Thermo Fisher, Carlsbad, CA). Proteins were concentrated and flash frozen in liquid nitrogen and stored at -80°.

FRET-based dissociation assay of the Cdc42:RhoGDI complex

Dual-labeled complexes of Alexa647-RhoGDI1 and Cy3-Cdc42 (wt or mutants) were formed *in situ* by mixing both proteins at final concentrations of 10 µM in Assay Buffer (50 mM HEPES pH=7.5, 50 mM NaCl, 2 mM $MgCl_2$, 0.1 mM GDP, 10 mM β-ME, 100 mg/ml β-casein) and incubating at RT for 10 minutes. Complexes were then diluted to 80 nM in Assay Buffer and transferred into a UV cuvette in a spectrofluorimeter (PTI QM-6) under constant excitation at 530 nm and monitoring of donor (λ = 570 nm) and acceptor (λ = 680 nm) fluorescence. After observation of stable baseline fluorescence emission for both acceptor and donor, 5 µM unlabeled RhoGDI1 (62-fold excess over Alexa647-RhoGDI1) was added at t = 0 s.

FRET-based association assay of Cdc42:RhoGDI complex formation

Complex formation between Cy3-labeled, prenylated Cdc42 (200nM) and Alexa647-labeled RhoGDI (790 – 2500nM) was measured in Assay Buffer after rapid mixing in a stopped-flow apparatus (Applied Photophysics) under constant excitation at 520 nm. A >3-fold excess of RhoGDI1 was maintained at all conditions to ensure pseudo-first order conditions. Time-dependent changes in sensitized emission were monitored with a 670/40nm bandpass filter.

Fluorescence-based nucleotide exchange assay of Cdc42

Mant-GDP-bound, prenylated Cdc42 was diluted in Assay Buffer to a final concentration of 100 nM in the presence or absence of excess RhoGDI1 (5 µM, either wildtype or variants) and excess unlabeled GDP (100 µM). The mix was transferred UV cuvette in a spectrofluorimeter (PTI QM-6) under constant excitation at 355 nm and monitoring of Mant fluorescence emission at 440 nM. After observation of stable baseline fluorescence emission, excess EDTA (10mM) was added at *t* = 0 s to initiate nucleotide exchange, which was evident by the decrease in Mant fluorescence due to solvent exposure of the labelled nucleotide.

Data analysis of fluorescence-based assays

To obtain the dissociation rate constant of the Cdc42:RhoGDI complex, we first calculated the ratio of acceptor over donor fluorescence intensity. We then normalized the data by subtracting the ratiometric signal at saturation after excess unlabeled RhoGDI1 addition and dividing by the initial ratiometric signal before RhoGDI1 addition. Dissociation rate constants were then determined by fitting time courses of normalized ratiometric signal by a mono-exponential decay function in Origin 9.0:

$$I(t) = e^{-k_{-GDI}\, t}$$

with I(t) being the normalized ratiometric signal as function of time and k_{-GDI} being the dissociation rate constant.

To determine apparent rates of complex formation between Alexa647-RhoGDI and prenylated Cy3-Cdc43, we first normalized the measured sensitized emission data by subtracting the initial sensitized emission signal before RhoGDI1 addition and dividing by the sensitized emission signal at saturation. For each RhoGDI concentration, we averaged time courses of the normalized sensitized emission data from five technical replicates. This averaged data was fitted by a mono-exponential association function in Origin 9.0:

$$I(t) = 1 - e^{-k_{obs}\, t}$$

with I(t) being the normalized sensitized emission signal as function of time and k_{obs} being the apparent rate of complex formation. Average rates of complex formation were then plotted as a function of the total Alexa647-RhoGDI concentration and fitted by a linear function in Origin 9.0:

$$k_{obs}([GDI]) = k_{-GDI} + k_{+GDI}\,[GDI]$$

with k_{obs}([GDI]) being the apparent rates of complex formation as function of the total GDI concentration [GDI], while k_{-GDI} and k_{+GDI} are the dissociation and association rate constants of the

RhoGDI:Cdc42 complex, respectively. k_{-GDI} was fixed to the independently measured value as described above.

Equilibrium dissociation constants (K_D) were calculated by dividing the measured dissociation rate constants for each RhoGDI1 variant by the association rate constant measured for wildtype RhoGDI1. We assume that the mutations in RhoGDI1 do not strongly affect the latter, because it falls close to the diffusion limit and can therefore only decrease.

To determine rates of nucleotide exchange, we first normalized fluorescence intensity time courses by subtracting the Mant fluorescence signal at saturation after EDTA addition and dividing by the initial Mant fluorescence signal before EDTA addition. This normalized data was fitted by a mono-exponential decay function in Origin 9.0:

$$I(t) = e^{-k_{-GDP}\,t}$$

with I(t) being the normalized fluorescence signal as function of time and k_{-GDP} being the nucleotide exchange rate.

NMR resonance assignment and data analysis

All NMR spectra were recorded at 298 K on a Bruker Avance NEO spectrometer operating at 800 MHz ^{1}H Larmor frequency, equipped with a CP TCI proton-optimized triple-resonance cryo probe. A suite of solution NMR (assignment) experiments were recorded, in particular 2D ^{15}N-^{1}H HSQC, 3D HNCA, 3D HNCO, 3D HN(CA)CO and HNCACB/CBCANH for each sample. For the isolated N-terminal samples 3D ^{15}N NOESY-HSQCs with a mixing time of 200 ms and 3D HNHA experiments with transfer time of 13.05 ms were recorded in addition. The *de novo* assignment was carried out manually using ccpNMR v3[3]. All NMR chemical shifts have been deposited into the BMRB under accession code 51835.

^{15}N longitudinal (R_1) and transverse (R_2) relaxation rates (in the presence of a CPMG pulse train) were measured in an interleaved manner, using a recycle delay of 1.5 s between experiments and the following delays for T_1: 10, 50, 100, 200, 300, 400, 500, 600, 800, 1020, 200 and 600 ms; and T_2: 10, 30, 50, 70, 90, 110, 130, 150, 170, 190, 50, 110 and 170 ms. Two and three experiments were repeated, respectively, to determine an experimental error of the measurements. Heteronuclear steady-state ^{15}N{^{1}H} NOE (hetNOE) spectra were acquired with two different data sets, one recorded without an initial proton saturation and the other with an initial proton saturation period of 3 s.

Residual dipolar couplings, ^{1}H/^{15}N-RDCs, for the apo full-length RhoGDI sample and the isolated N-terminus (1-69) were collected using Pf1 filamentous phage (purchased from Asla Biotech) with a concentration of 15 mg/mL (fl) or 29 mg/mL (N-term), respectively, as an alignment medium, resulting in a $^{2}D_2O$ quadrupolar splitting of 14 (fl) and 25 Hz (N-term), respectively. The RDCs were determined via IPAP HSQC experiments.[4]

CheSPI analysis[5] of (residual) secondary structure was run using the Web server (https://st-protein.chem.au.dk/chespi). The software was provided with an nmr-star v2.1 file containing the protein sequence, experimental conditions (7.5 pH , 0.15 M ionic strength, 25°C), and the experimental chemical shift information for $^{1}H^{N}$, N, CO, Cα, and Cβ. H^{α} information was included for the isolated N-terminal samples. Secondary chemical shifts were calculated with respect to neighbor-corrected random coil chemical shifts using using the Web server (https://st-protein.chem.au.dk/chezod) as described in Nielsen et al.[6] Chemical-shift perturbations were calculated as $(\Delta\delta^2\,(^{1}H^{N}) + \Delta\delta^2\,(^{13}C^{\alpha})/16 + \Delta\delta^2\,(^{13}C^{\beta})/16 + \Delta\delta^2\,(^{15}N)/100)^{0.5}$.

Molecular-dynamics simulations

We performed structure-based model (SBM) simulations as a first access to interrogate GDI structural properties. Structure-based simulations – also known as Go-like models – are known to capture the mechanistic aspects of protein folding at a fraction of the computational cost of conventional all-atom simulations, since the protein's native structure is directly encoded into the forcefield. Starting from the structure of RhoGDI in complex with Rho (PDBID: 1DOA[7]), we used SMOG2[8] to build an all-atom Lennard-Jones SBM[9] for RhoGDI, using a shadow map definition for native contacts[10] with default parameters. Simulations were performed in GROMACS 2022[11], using a stochastic integrator with a time constant of 1 ps and a 2 fs time step. All simulations were run for 200,000,000 steps. We performed a total of 20 simulations, where 10 started from the fully folded state and 10 from a RhoGDI model with unfolded N-terminus. We used a temperature range between 0.9 – 1.1 times the melting temperature. Under these conditions, the core of RhoGDI remains properly folded during the entire simulation.

We used VMD[12] to analyze the number of native contacts along the trajectories. We considered a contact to be formed if the two atoms were at a distance smaller than 1.2 times the one observed in the reference structure. The free energy profiles associates with the folding of the N-terminus were obtained using the weighted-histogram analysis method[13] as implemented in the SMOG package.

Supplementary Figures:

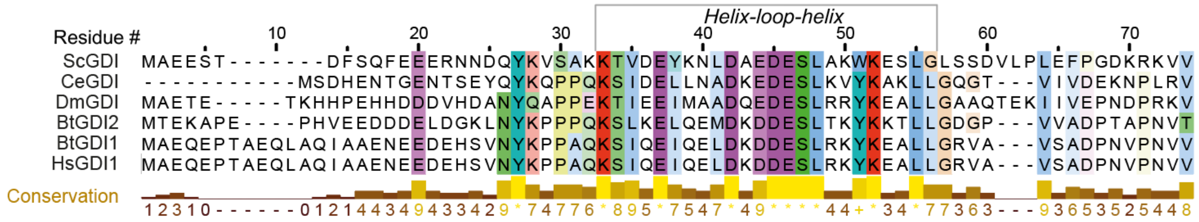

Fig. S1: Sequence alignment of the N-terminal region of different RhoGDI orthologs and isoforms according to Clustal, adding more details to main text Fig. 1C. The conservation score was calculated according to the AMAS method of multiple sequence alignment analysis[14]. Sc: *Saccharomyces cerevisiae*, Ce: *Caenorhabditis elegans*, Dm: *Drosophila melanogaster*, Bt: *Bos taurus*, Hs: *Homo sapiens*. Color depicts BLOSUM62 score. Val74 marks the start of the C-terminal Ig-like domain.

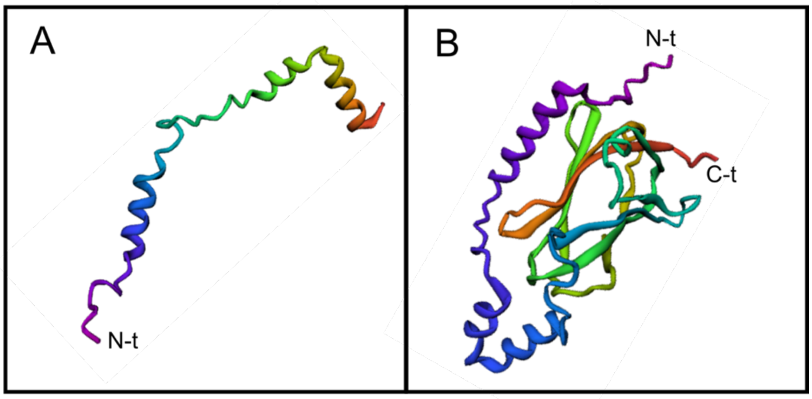

Fig. S2: Robetta[15] prediction of the secondary structure for the isolated N-terminal domain **(A)** and the full-length RhoGDI1 **(B)**.

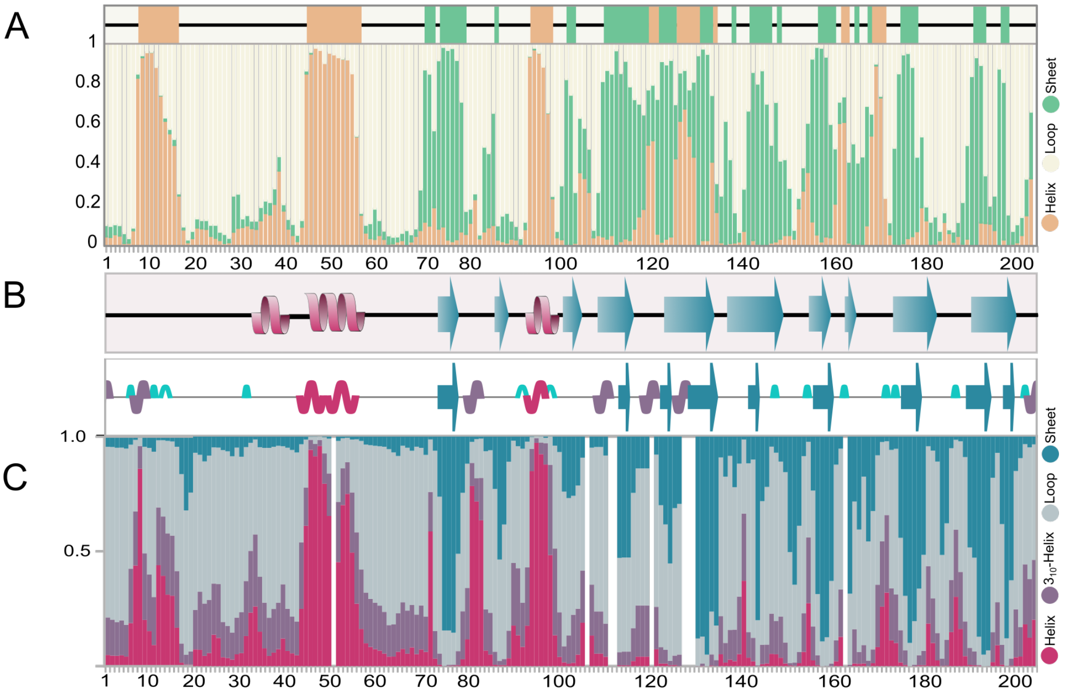

Fig. S3: Secondary structure prediction of full-length RhoGDI from experimental chemical-shift information. **A)** Talos+ prediction[16] using HN, Cα, Cβ, CO, and N chemical shift with the representation of the proposed secondary structure. **B)** X-ray structure of the complex RhoGDI1 with Cdc42 from PDB 1DOA. **C)** Prediction of secondary structure by CheSPI[5] using HN, Cα, Cβ, CO, and N chemical shift, represented as relative propensity (bottom) and visualization of most likely structure above it.

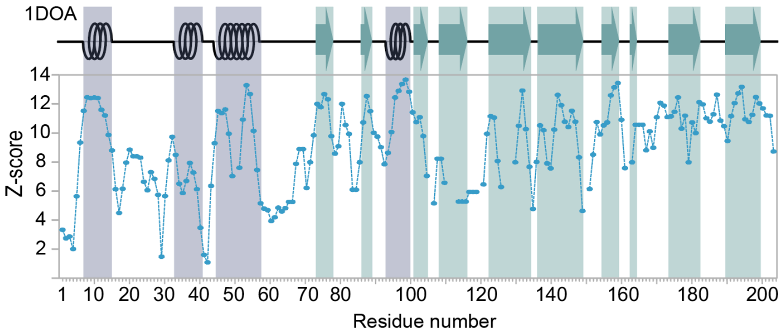

Fig. S4: Z- score of apo RhoGDI1 full length from CheSPI. A low Z-score value indicates a disordered region, while a high value indicates the presence of more stably adopted secondary structure. For an intrinsically disordered N-terminus, a low Z-score would be expected. Instead, the scores confirm the presence of pronounced secondary structure within the N-terminus of the apo form of the full-length protein.

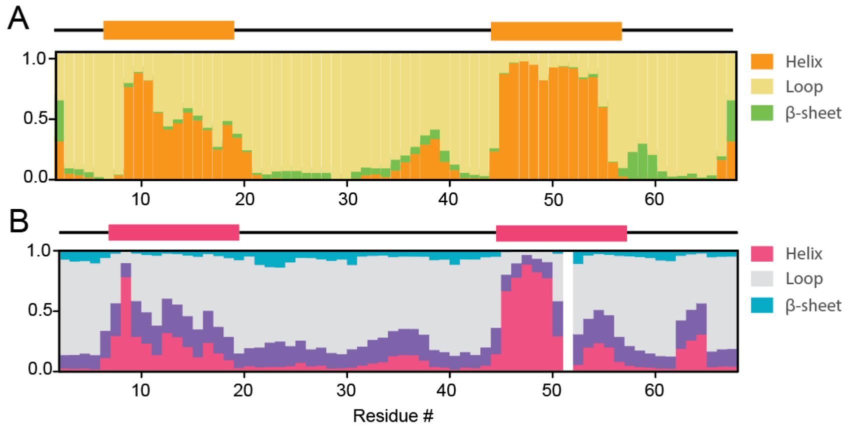

Fig. S5: Predictions of the secondary structure based on experimental chemical shifts for the isolated residues 1-69. **A)** Talos+ prediction[16] using H^N, H^α, C^α, C^β, CO, and N chemical shift with the representation of the proposed secondary structure. **B)** Prediction of secondary structure by CheSPI [5] using H^N, H^α, C^α, C^β, CO, and N chemical shift.

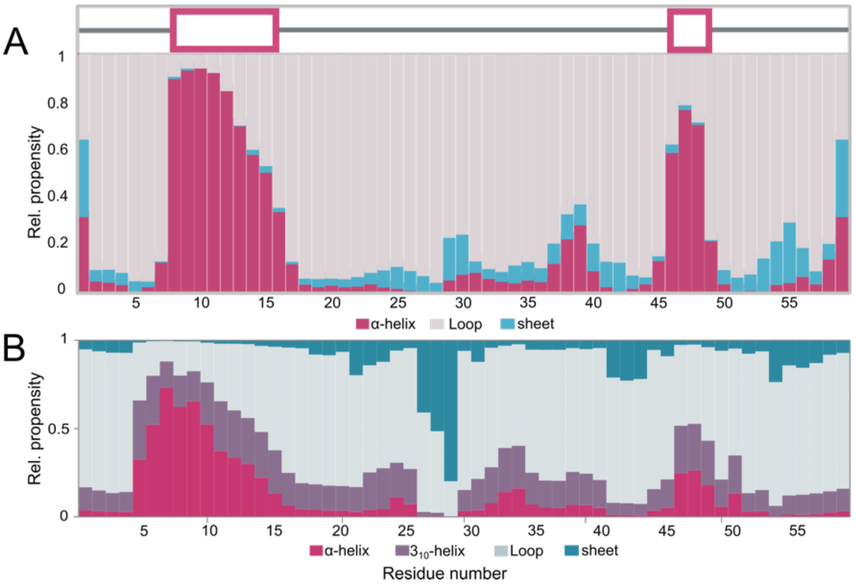

Fig. S6: Predictions of the secondary structure based on experimental chemical shifts for the isolated residues 1-59. **A)** Talos+ prediction[16] using H^N, H^α, C^α, C^β, CO, and N chemical shift with the representation of the proposed secondary structure. **B)** Prediction of secondary structure by CheSPI [5] using HN, H^α, C^α, C^β, CO, and N chemical shift. Note the changes in temporary secondary structure not only at the helix-loop-helix motif (despite the shorter construct exceeding the C-terminal transient helix by five residues) but also for the first transient helix (8-15).

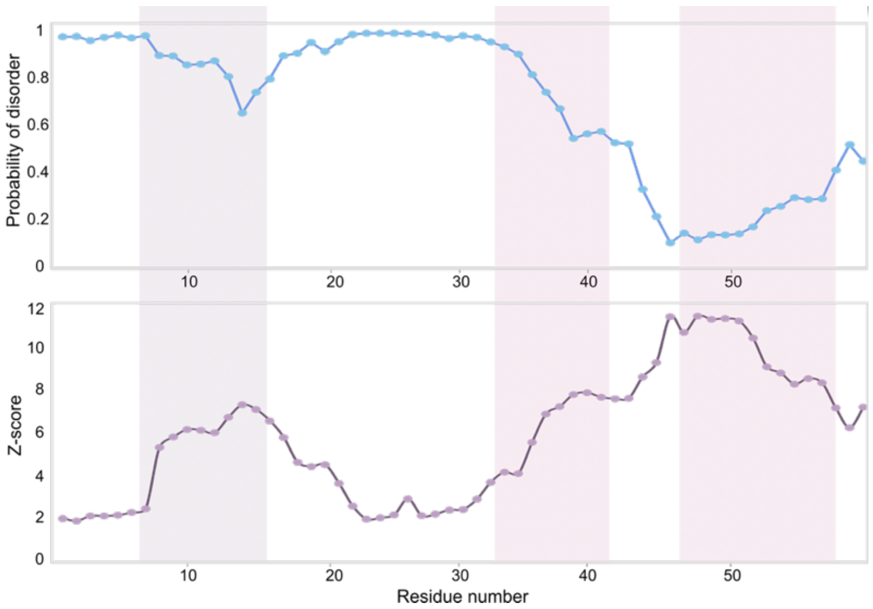

Fig. S7: ODiNPred[17] calculation of the probability of disorder based of the isolated N-terminal residues 1-59 (top) and CheSPI[5] Z-score of the isolated N-terminal domain of RhoGDI (bottom). Purple shades denote helical stretches found in the X-ray structure 1DOA.

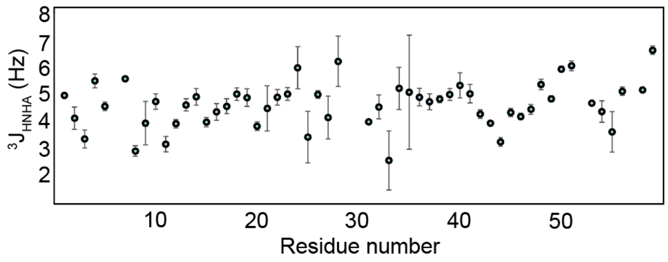

Fig. S8: $^3J^{HNHa}$ couplings of the shorter isolated N-terminal sample (residues 1-59), consistently small values (<~4.5 Hz) being typical for helical stretches. The trends of this construct are reduced compared to the longer N-terminus (shown in Main Text Fig. 4F), consistent with chemical-shift-based residual secondary-structural propensities of this construct (compare Fig. S6).

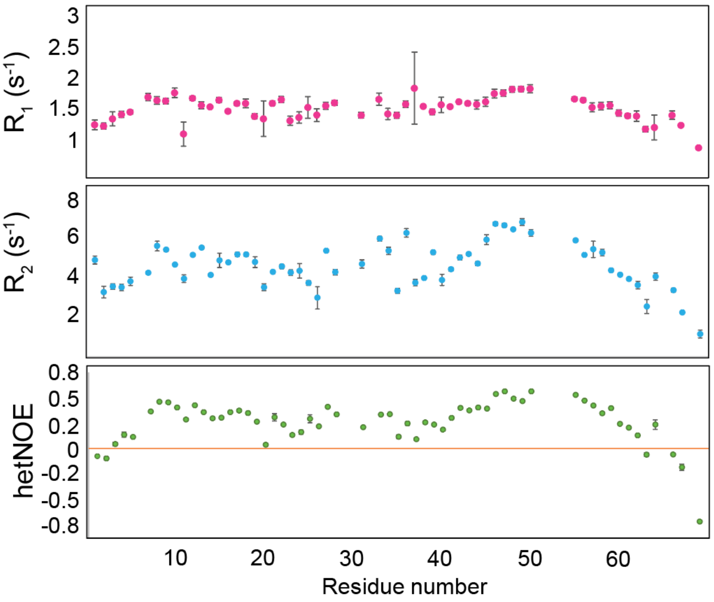

Fig. S9: R_1, R_2, and $^{15}N\{^1H\}$ NOE (hetNOE) data for the longer isolated N-terminus (residues 1-69).

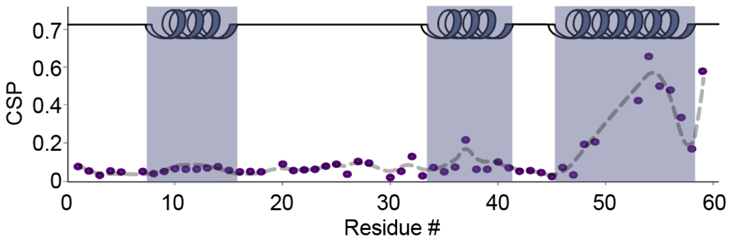

Fig. S10: Chemical-shift perturbations between the shorter isolated N-terminus (residues 1-59) and the full-length protein as a function of residue. The CSPs are in line with the longer N-terminal sample (residues 1-69, see Fig. S11) roughly up to residue 50. Here, however, strongest CSPs occur for residues 53-57, in line with a reduction of helicity also in the second helix of the helix-loop-helix motif for this construct (compare Figs. S5/S6). The trendline has been included to discern the consistent trend for this helix (*decreasing* again towards the end of the helix) and the boundary effect for the last residue with an expected, large CSP *increase* due to the chemical difference associated with being the new C-terminus. The top row denotes crystallographic secondary structure (PDB 1DOA).

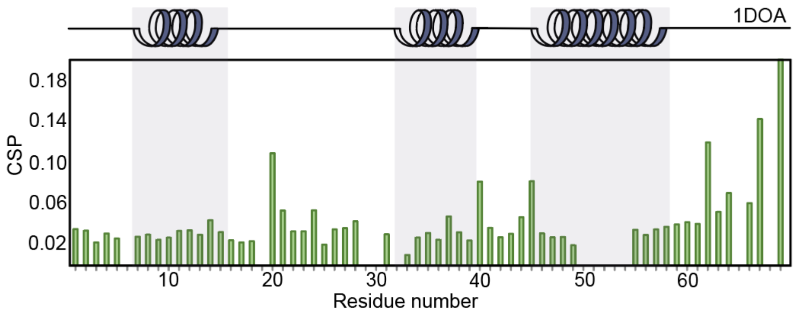

Fig. S11: Chemical-shift perturbations between the longer isolated N-terminus (1-69) and the full-length protein as a function of residue. (Also see Fig. S10 above.) Top row: secondary structure in the X-ray structure of the complex (PDB 1DOA).

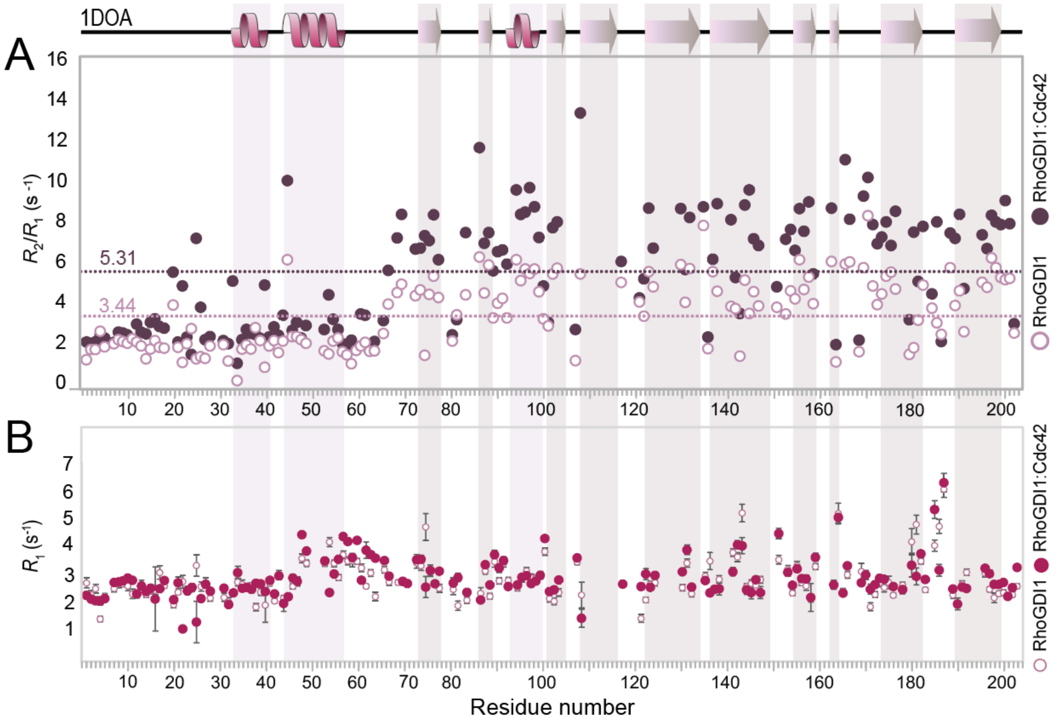

Fig. S12: ^{15}N R_2/R_1 ratio (**A**) and R_1 rates (**B**) for RhoGDI1 (open circles) and RhoGDI1 in complex with Cdc42 (dark circles), where the dashed lines in A) indicate the overall average R_2/R_1 ratio value for the protein apo (light purple) and the complex (dark purple) excluding 10% outliers.

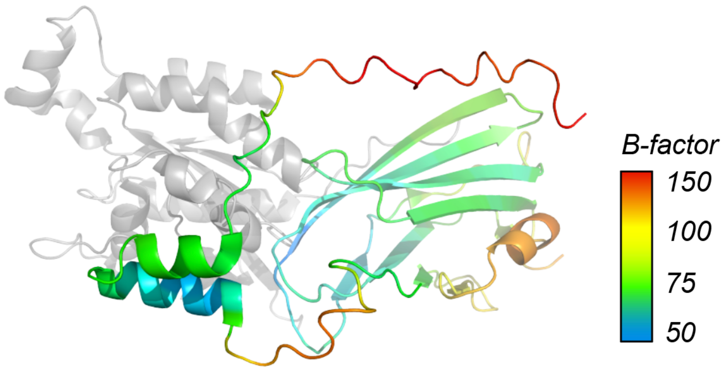

Fig. S13: C^{α} temperature B-factors seen in the RhoGDI1:Cdc42 complex (PDB: 1DOA), represented on the structure by the color code shown on the right. Cdc42 is shown in gray.

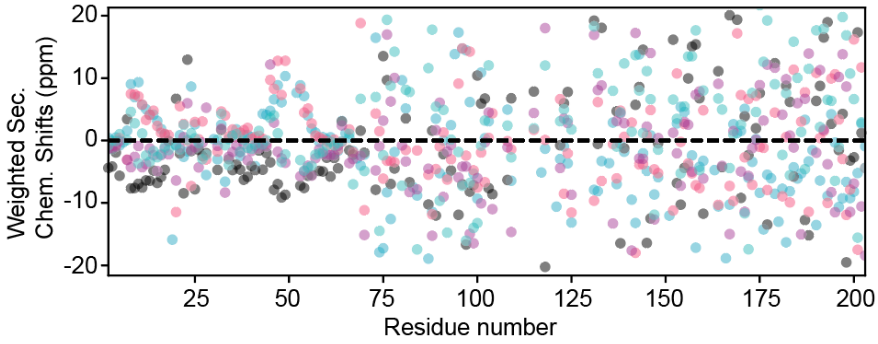

Fig. S14: Individual secondary chemical shifts ($\delta_i - \delta_{rc}$) for the $^{1}H^{N}$, ^{15}N, $^{13}C^{\alpha}$, $^{13}C^{\beta}$, and ^{13}CO chemical shifts of the complex, drawn in cyan, magenta, red, black, and blue, respectively.

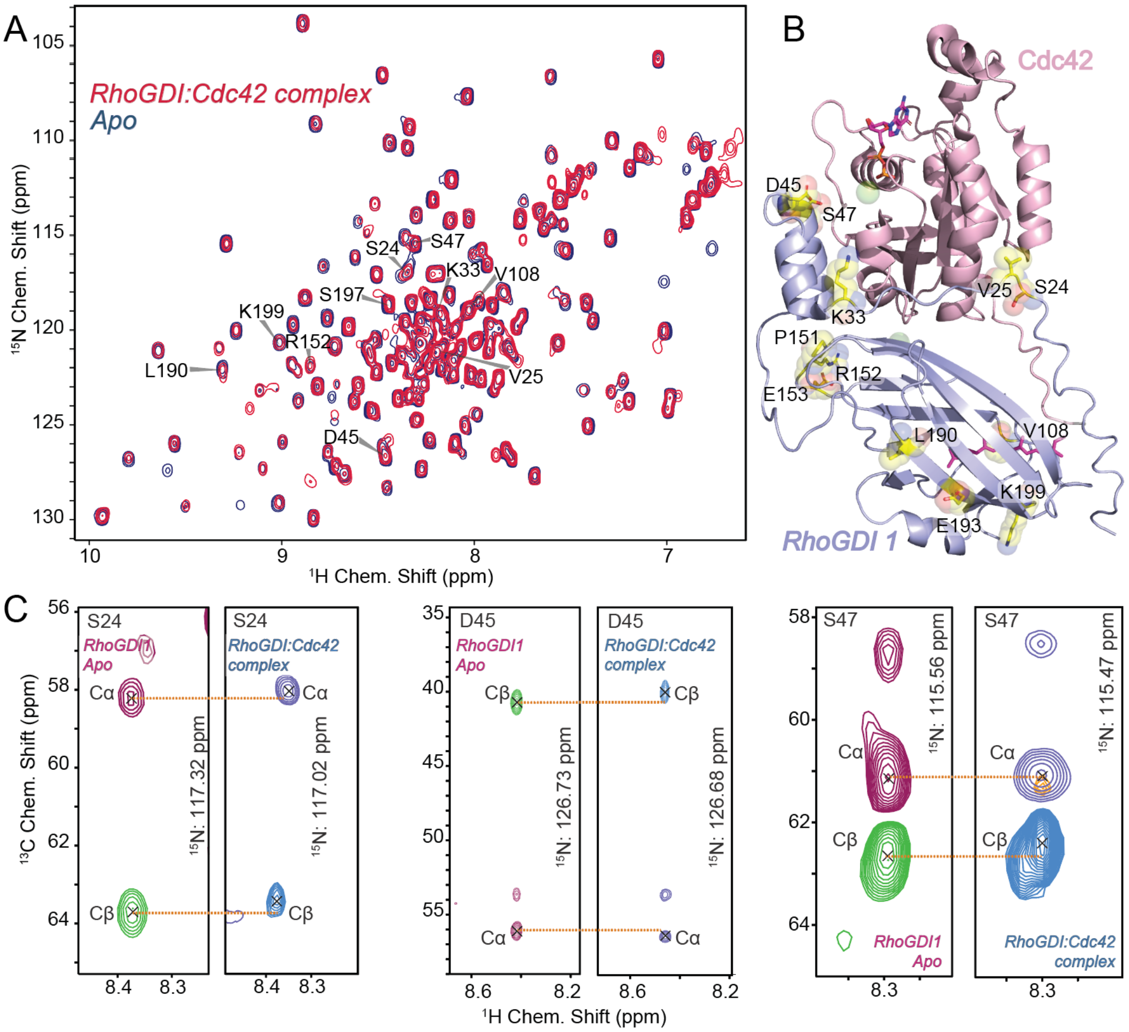

Fig. S15: Visualization of CSPs obtained in full-length RhoGDI upon complex formation with Cdc42. **A)** Overlay of HSQC spectra of apo RhoGDI (magenta) and RhoGDI in complex with Cdc42 (purple) with peak annotations of significant shifts. **B)** Visualization of residues with high CSPs in the crystallographic structure. **C)** Some strips extracted from CBCANH experiments for RhoGDI apo and RhoGDI in complex with Cdc42 that show perturbations of ^{13}C chemical shifts. The annotated residues are those with a CSP $(\Delta\delta^2\,(^1H^N) + \Delta\delta^2\,(^{13}C^{\alpha})/16 + \Delta\delta^2\,(^{13}C^{\beta})/16 + \Delta\delta^2\,(^{15}N)/100)^{0.5}$ larger than two times the standard deviation (i. e., using a cutoff value of 0.04).

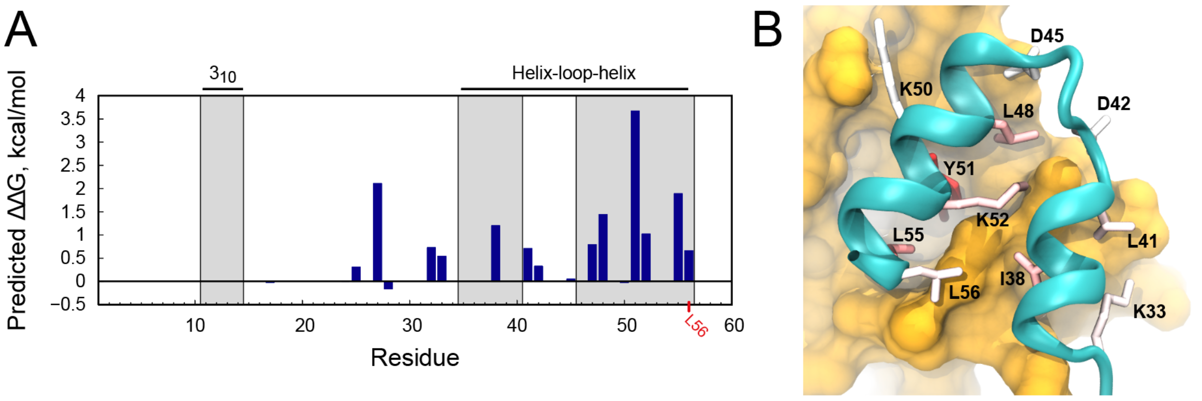

Fig. S16: Alanine scanning of the interface between Cdc42 and RhoGDI1. **A)** Calculated ΔΔG for the first 60 amino acids of RhoGDI. The gray boxes highlight regions with helical structure. **B)** Predicted ΔΔG mapped into the structure of the helix-loop-helix motif. The amino acids are colored according to the predicted change of affinity when mutated to alanine, with white being 0 and the intensity of red reflecting increasing loss of affinity.

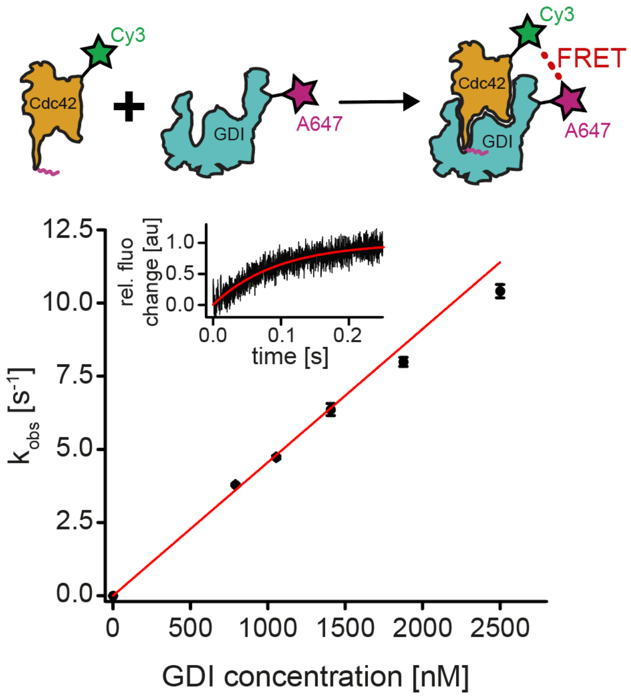

Fig. S17: Top: Scheme of the FRET-based association experiment. Cy3-labeled, prenylated Cdc42 (100 nM) was mixed in a stopped-flow spectrometer with various excess concentrations of Alexa647-labeled RhoGDI1 (790 – 2500 nM). Association of the complex was followed over time by the gain in FRET between the two fluorophores. Bottom: Observed association rates (dots) as a function of the total Alexa647-RhoGDI1 concentration. The value at c = 0 µM was fixed to the independently measured dissociation rate constant (k_{-GDI}, Main Text Fig. 6 B,C). A linear fit to the data (red) yields the association rate constant (k_{+GDI}, Main Text Fig. 6C). Inset: Raw time trace of fluorescence-sensitized emission (black) at 2500 nM Alexa647-RhoGDI1 and a mono-exponential fit to the data (red).

References:

1. Gavriljuk K, Itzen A, Goody RS, Gerwert K, Kötting C. Membrane extraction of Rab proteins by GDP dissociation inhibitor characterized using attenuated total reflection infrared spectroscopy. *Proc Natl Acad Sci U S A* **110**, 13380-13385 (2013).

2. Golding AE, Visco I, Bieling P, Bement WM. Extraction of active RhoGTPases by RhoGDI regulates spatiotemporal patterning of RhoGTPases. *Elife* **8**, e50471 (2019).

3. Skinner SP, Fogh RH, Boucher W, Ragan TJ, Mureddu LG, Vuister GW. CcpNmr AnalysisAssign: a flexible platform for integrated NMR analysis. *J Biomol NMR* **66**, 111-124 (2016).

4. Ottiger M, Delaglio F, Bax A. Measurement of J and Dipolar Couplings from Simplified Two-Dimensional NMR Spectra. *J Magn Reson* **131**, 373-378 (1998).

5. Nielsen JT, Mulder FAA. CheSPI: chemical shift secondary structure population inference. *J Biomol NMR* **75**, 273-291 (2021).

6. Nielsen JT, Mulder FA. There is Diversity in Disorder-"In all Chaos there is a Cosmos, in all Disorder a Secret Order". *Front Mol Biosci* **3**, 4 (2016).

7. Hoffman GR, Nassar N, Cerione RA. Structure of the Rho Family GTP-Binding Protein Cdc42 in Complex with the Multifunctional Regulator RhoGDI. *Cell* **100**, 345-356 (2000).

8. Noel JK*, et al.* SMOG 2: A Versatile Software Package for Generating Structure-Based Models. *PLoS Comput Biol* **12**, e1004794 (2016).

9. Whitford PC, Noel JK, Gosavi S, Schug A, Sanbonmatsu KY, Onuchic JN. An all-atom structure-based potential for proteins: bridging minimal models with all-atom empirical forcefields. *Proteins* **75**, 430-441 (2009).

10. Noel JK, Whitford PC, Onuchic JN. The shadow map: a general contact definition for capturing the dynamics of biomolecular folding and function. *J Phys Chem B* **116**, 8692-8702 (2012).

11. Abraham MJ*, et al.* GROMACS: High performance molecular simulations through multi-level parallelism from laptops to supercomputers. *SoftwareX* **1-2**, 19-25 (2015).

12. Humphrey W, Dalke A, Schulten K. VMD: visual molecular dynamics. *J Mol Graph* **14**, 33-38, 27-38 (1996).

13. Kumar S, Rosenberg JM, Bouzida D, Swendsen RH, Kollman PA. THE weighted histogram analysis method for free-energy calculations on biomolecules. I. The method. *J Comp Chem* **13**, 1011-1021 (1992).

14. Livingstone CD, Barton GJ. Protein sequence alignments: a strategy for the hierarchical analysis of residue conservation. *Comput Appl Biosci* **9**, 745-756 (1993).

15. Kim DE, Chivian D, Baker D. Protein structure prediction and analysis using the Robetta server. *Nucleic Acids Res* **32**, W526-531 (2004).

16. Shen Y, Delaglio F, Cornilescu G, Bax A. TALOS+: a hybrid method for predicting protein backbone torsion angles from NMR chemical shifts. *J Biomol NMR* **44**, 213-223 (2009).

17. Dass R, Mulder FAA, Nielsen JT. ODiNPred: comprehensive prediction of protein order and disorder. *Sci Rep* **10**, 14780 (2020).
